# Clinical efficacy and imaging analysis for the surgical treatment of thoracolumbar infections in elderly patients: a retrospective study

**DOI:** 10.1038/s41598-023-36985-6

**Published:** 2023-06-26

**Authors:** Yanlong Zhong, Benyu Tang, Zizhen Zhang, Yonghong Sheng, Chao Li, Jia Guo, Shiwei Luo, Haoqun Yao, Zongmiao Wan

**Affiliations:** 1 Department of Orthopedics, The First Affiliated Hospital of Nanchang University, 17 Yongwai Street, Nanchang, 330006 Jiangxi People’s Republic of China; 2 Department of Orthopedics, The People’s Hospital of Yi Chun City, Yichun, 336000 Jiangxi People’s Republic of China

**Keywords:** Infectious diseases, Neurological disorders, Outcomes research

## Abstract

Few reports have been conducted to comparing surgical results and safety evaluations between the different types of infections in geriatric patients with thoracolumbar infections. The aim of this study is to investigate the safety and efficacy of surgical treatment for thoracolumbar infections in elderly patients. 21 patients with pyogenic spondylodiscitis (PS) and 26 patients with tuberculous spondylodiscitis (TS) were enrolled in the study. All patients were treated using one-stage posterior debridement, decompression, and pedicle screw fixation. Comparison of operative safety parameters between the two groups. Clinical efficacy was evaluated using visual analogue scale (VAS) score, the American Spinal Injury Association (ASIA) grade, the short form (SF)-36 survey and Oswestry disability index (ODI) to determine patient quality of life pre- and post-operatively. Hospitalisation and intensive care unit duration in the PS group were significantly shorter than in the TS group (P < 0.05). The total incidence of post-operative complications for both groups was 44.7%. More complications occurred in the TS group, but the difference was not significant. The scores of VAS, ODI and SF-36 of all 47 patients were significantly improved compared with those before operation.The VAS and SF-36 scores (physical component) were significantly better in the PS group 6 months post-operatively, and the SF-36 (mental component) scores were significantly better in the PS group at the 1-year follow-up. Neurological status in both groups improved post-operatively, and 83% of patients reported satisfactory results based on the modified MacNab standard. Imaging results showed that bone graft fusion improved in both groups at 6 months, 1 year and at the final follow-up. One-stage posterior debridement, decompression, interbody fusion, and internal fixation can be considered a safe and effective method of treating spinal infections in the elderly. This method can improve nerve function, reconstruct spinal stability, and enhance the quality of life of elderly patients. Both PS and TS who underwent surgery achieve similar clinical and radiological results.

## Introduction

The incidence of spinal infections is on the rise as people live longer, diagnosis and treatment techniques improve, and chronic wasting diseases, invasive spinal procedures, spinal implants, intravenous drug abuse, and immunodeficiency diseases increase^1,2^. Infectious spondylitis is a special biological type of infectious disease, which affects the vertebral body, intervertebral disc, and adjacent paravertebral soft tissue^3,4^. During the development of a spinal infection, abscesses or edemas form that destroy the vertebrae or cause neurological disorders^5^. The incidence of spinal infection is often hidden. If not diagnosed and treated in time, severe cases can lead to spinal deformity, neurological impairment, paralysis, and even death. A variety of pathogens can cause spinal infections, with ordinary bacteria and *Mycobacterium tuberculosis* being the primary pathogens^6^. Pyogenic spondylodiscitis (PS) is an infectious spondylitis commonly caused by staphylococcus species and viridians streptococci, while spondylitis caused by *M. tuberculosis* is termed tuberculous spondylodiscitis (TS)^5,7^.

For elderly patients with decreased immune resistance and poor physical functioning, conservative treatments such as immobilisation and antibiotic administration should be the first choice^8,9^. However, these treatments require prolonged bed rest, which can lead to complications including urinary system infections, lower limb deep venous thrombosis and pulmonary infection, all of which may seriously affect patient quality of life^10^. Abscesses, or edema, occur during the course of the disease and can form to destroy the vertebrae or cause neurological deficits^7^. Therefore, to optimally target the pathology of the disease and facilitate early mobilisation in the elderly, a quick, effective, and safe approach is needed. Surgical decompression is the preferred treatment when neurological deficits or progressive deformity occurs during disease progression^11–15^.

Currently, the surgical treatment for infectious spondylitis is divided into the following four techniques, including posterior stabilization alone, anterior only, anterior combined, or posterior combined^16,17^. Several authors have reported the results for these surgical techniques and have confirmed that the single posterior approach is well established in patients with infectious spondylitis^18,19^. However, there are few reports comparing surgical results and safety evaluations between the different types of infections in geriatric patients with thoracolumbar infections^10,20^. In this study, we investigated the clinical and radiological results of one-stage posterior surgery for elderly patients with PS or TS at a single institution, and evaluated the efficacy and safety of the surgical treatment.

## Materials and methods

### Patient characteristics

We used our hospital electronic medical record system (Haitai^®^ 3.0, Nanjing, China) and image archiving and communication system (Carestream^®^, Jiangxi, China) to retrospectively analyse the data of 59 elderly patients with infectious spondylitis who underwent opening one-stage posterior debridement, decompression, and pedicle screw fixation at our department from January 2012 to September 2017. The inclusion criteria were: (1) patients who were diagnosed with suppurative or tuberculous spondylitis and had received surgical treatment, (2) age over 65-years-of-age at the time of operation, and (3) had a post-operative follow-up of ≥ 1 year. The exclusion criteria were: (1) incomplete medical records or imaging data, and (2) patients without telephones or out-patient follow-up. The diagnosis of infectious spondylitis was based on clinical symptoms, laboratory results and radiological evidence. Histopathology following debridement was confirmed. Patients were divided into a PS or TS group according to the pathological results (Tables 1, 2).

**Table 1:** Demographic data of PS group.

Case	Age (year)/sex	Drinking	Somke	Affected level	Concomitant diseases	IDA	CU	ASA	Indications of surgery	Ogranism/pathology	Follow-up period (months)
1	76/M	NO	YES	L1–L2	OST	NO	NO	I	B, E	Negative/PI	23
2	67/M	NO	NO	L4–L5	HT, DM, MAL	NO	NO	III	A, B, E	MASA/PI	36
3	74/F	YES	YES	L3–L4	CD, DM	NO	NO	II	A, C, E	*S. aureus*/PI	16
4	74/M	NO	NO	T8–T9	CD, PD, MAL	NO	YES	III	A, B, E	Negative/PI	24
5	79/F	NO	NO	L2–L3	OST	NO	NO	I	A, B, E	*B. cepacia*/PI	13
6	70/M	NO	YES	L1–L3	DM, HEP	NO	NO	II	A, C, E	*S. aureus*/PI	27
7	74/F	YES	NO	T8–T9	CD, HEP	NO	NO	II	A, E	MASA/PI	25
8	76/M	NO	NO	T9–T10	HT, OST	NO	YES	II	A, B, E	*E. coli*/PI	27
9	76/M	YES	NO	T6–T7	RA, HEP	NO	NO	II	B, E	Negative/PI	45
10	67/M	NO	NO	L3–L4	CD, PD	NO	NO	II	A, E	*S. aureus*/PI	44
11	78/F	YES	NO	L4–L5	CI, PD	NO	NO	III	A, E	Negative/PI	14
12	78/M	NO	NO	L3–L4	HT, DM	NO	YES	II	A, C, E	*S. aureus*/PI	21
13	75/F	NO	YES	T9–T10	CD, OST	NO	NO	II	B, E	Negative/PI	24
14	82/F	YES	YES	L3–L4	PD, MAL	NO	NO	III	B, E	Negative/PI	18
15	74/F	NO	YES	T11–T12	–	NO	YES	I	B, E	Negative/PI	24
16	72/M	YES	No	T12–L1	RF, DM, MAL	NO	NO	III	A, B, E	*S. aureus*/PI	36
17	69/F	NO	YES	T8–T9	MAL	NO	NO	I	B, E	Negative/PI	18
18	73/F	YES	YES	T11–T12	CD, HT	NO	NO	III	A, E	Negative/PI	24
19	78/M	NO	YES	L4–L5	OST	NO	NO	II	B, E	Negative/PI	30
20	78/F	NO	YES	T11–T12	–	NO	NO	I	A, E	Negative/PI	24
21	79/F	NO	YES	L2–L3	DM, CD, HT	NO	NO	II	B, E	Negative/PI	34

*TS* Tuberculous spondylodiscitis, *ASA* American Society of Anesthesiologists, *IDA* Intravenous drug abuse, *CU* Corticosteroid use, *OST* Osteoporosis, *HT* Hypertension, *DM* Diabetes mellitus, *MAL* Malignancy, *CD* Cardiac disease, *HEP* Hepatitis, *RA* Rheumatoid arthritis, *PD* Pulmonary disease, *RF* Renal failure, *PI* Pyogenic inflammation.

Indications for surgery: *A* Severe pain, *B* Deteriorating or severe neurological deficit, *C* Severe deformity, *D* Spinal instability, *E* Persistent infection that does not respond to conservative treatment.

**Table 2:** Demographic data of TS group.

Case	Age (year)/sex	Drinking	Smoke	Affected level	Concomitant diseases	IDA	CU	ASA	Indications of surgery	Ogranism/pathology	Follow-up period (months)
1	79/M	NO	No	L3–L4	HT, DM	NO	NO	II	B, E	Negative/GI	12
2	68/M	NO	No	L4–L5	CD, PD	NO	NO	I	A, B, E	Negative/GI	68
3	66/M	YES	No	L2–L3	HT, DM	NO	NO	II	B, E	Negative/GI	14
4	68/M	NO	No	T12–L1	PD, HEP	NO	NO	II	B, E	Negative/GI	23
5	76/F	YES	No	L4–L5	CD, OST	NO	NO	II	A, B, E	Negative/GI	12
6	79/M	YES	No	L4–L5	DM, HEP	NO	YES	III	A, E	Negative/GI	12
7	76/M	NO	No	L1–L3	CI, OST	NO	NO	II	A, B, D, E	Negative/GI	17
8	70/M	NO	No	L2–L3	HEP, OST	NO	NO	III	B, E	*T. bacillus*/GI	26
9	73/F	YES	Yes	L3–L5	CD, HT	NO	NO	II	A, B, C, E	Negative/GI	29
10	71/F	NO	No	L4–L5	CD, MAL	NO	NO	I	A, E	Negative/GI	28
11	76/F	NO	Yes	L4–L5	PD, MAL	NO	NO	II	A, B, E	Negative/GI	13
12	72/M	YES	No	L3–L4	PD	NO	YES	II	B, E	Negative/GI	14
13	78/M	NO	No	T9–T10	DM, PD	NO	NO	II	B, E	Negative/GI	45
14	78/M	NO	No	L4–L5	HT, PD	NO	NO	I	A, B, E	Negative/GI	36
15	78/F	YES	No	L2–L3	OST	NO	NO	II	A, E	Negative/GI	35
16	77/F	NO	No	T11–T12	HT, CI	NO	NO	II	A, E	Negative/GI	31
17	80/M	YES	No	T11–T12	MAL	NO	YES	I	A, B, E	Negative/GI	31
18	77/F	NO	NO	T11–T12	PD, HEP	NO	NO	II	A, E	Negative/GI	46
19	77/M	YES	Yes	L3–L4	RA	NO	NO	III	A, B, E	Negative/GI	36
20	72/F	NO	No	T10–T11	DM	NO	NO	II	A, E	Negative/GI	29
21	74/F	NO	No	L2–L3	–	NO	NO	II	A, B, E	Negative/GI	58
22	76/M	NO	Yes	T7–T8	DM, HT, MAL	NO	NO	III	A, B, D, E	*T. bacillus*/GI	56
23	76/F	NO	No	T4–T6	HT, DM	NO	NO	II	B, C, E	Negative/GI	19
24	66/F	NO	No	L2–L3	–	NO	YES	I	C, D, E	*T. bacillus*/GI	50
25	68/M	NO	No	T5–T6	DM	NO	NO	I	C, E	Negative/GI	20
26	74/F	NO	No	L2–L3	HT	NO	NO	II	C, D, E	Negative/GI	44

*TS* Tuberculous spondylodiscitis, *ASA* American Society of Anesthesiologists, *IDA* Intravenous drug abuse, *CU* Corticosteroid use, *OST* Osteoporosis, *HT* Hypertension, *DM* Diabetes mellitus, *MAL* Malignancy, *CD* Cardiac disease, *HEP* Hepatitis, *RA* Rheumatoid arthritis, *PD* Pulmonary disease, *GI* Granulomatous inflammation.

Indications for surgery: *A* Severe pain, *B* Deteriorating or severe neurological deficit, *C* Severe deformity, *D* Spinal instability, *E* Persistent infection that does not respond to conservative treatment.

### Treatment process

Pre-operatively, patients with a positive blood culture were treated with antibiotics according to microbial sensitivity, and patients with a negative culture were administered empirical, broad-spectrum antibiotics or anti-tuberculosis therapy based on signs, symptoms, and imaging results. Preoperative anaesthesia and surgical tolerance evaluation were performed to ensure the safe operation.

Intraoperatively, posterior laminectomy and pedicle screw fixation were performed under general anaesthesia. As far as possible, pedicle screws should not be applied directly to the affected vertebra. When the infection of the vertebral body is severely damaged, fixation is done above or below the segment. After pedicle screw fixation, the intervertebral space was drawn to clear the infected endplate, disc, and soft tissue. After careful debridement, the space was filled with autogenous iliac bone grafts. All patients underwent tricortical iliac bone graft. After the implant was firmly secured with a compressed space, the posterolateral implant was performed to achieve a 360° fusion. Depending on the preoperative and intraoperative diagnosis, vancomycin (0.5 g) was used locally if suppurative inflammation was considered, and isoniazid (0.2 g) and streptomycin (0.1 g) were used locally if tuberculosis was considered.

Post-operatively, lesions were subjected to pathological examination and tissue culture. Patients with PS were given antibiotics according to microbial sensitivity. Regularly monitor laboratory parameters (leukocyte count, ESR and CRP), adjust antibiotic dosage according to laboratory parameters, and test drug levels if necessary. Patients with TS received standard anti-tuberculosis chemotherapy (300 mg day^−1^ isoniazid, 450 mg day^−1^ rifampicin, 750 mg day^−1^ ethambutol, 750 mg day^−1^ pyrazinamide) for 12–18 months following their operation. Usually, passive joint muscle rehabilitation exercises can be performed after the anesthesia dissipates. The next day after surgery, the patient was encouraged to be active and allowed to get out of bed and sit on the edge of the bed with a brace after the drainage tube was removed. During hospitalization, gait rehabilitation training was used to restore lower limb function. All patients wore plastic orthotics for 8 weeks after discharge for postoperative stabilization.

### Methods of using orthotics

Firstly, the patient should be in bed and turn over axially, turn to the side and maintain the lateral decubitus position. Put the posterior part of the thoracolumbar orthotics on the bed surface consistent with the position of the back, and then ask the patient to lie in the supine position, and press the posterior part of the thoracolumbar orthotics just below the body, which is completely consistent with the protective part. The posterior part of the thoracolumbar orthotics was first fixed to ensure stability, and then the anterior part of the thoracolumbar orthotics was placed on the patient's chest and abdomen, and the front and back parts of the thoracolumbar brace were fixed together to ensure stability.

### Peri-operative safety evaluations

Two groups of indexes related to surgical safety were reviewed and recorded in the medical records system including hospital-stay duration, operation time, blood loss, suspended red blood cell transfusion (peri-operative period) and duration in the intensive care unit (ICU). Common post-operative complications were closely observed and included incision infection, pulmonary infection, venous thrombosis, heart failure and liver function damage.

### Clinical efficacy evaluations

Clinical outcomes were assessed using visual analogue scale (VAS) to evaluate back pain. The Oswestry disability index (ODI) was used to quantify disability associated with lower back pain, and the short form (SF)-36 concise health status questionnaire was used to determine patient quality of life both pre-operatively and post-operatively (at the 3-month, 6-month, 1-year, and final follow-up time points). The American Spinal Injury Association (ASIA) scale was used to compare the state of the nervous system pre- and post-operatively in the two groups. Surgical outcome satisfaction was assessed using the modified MacNab criteria^21^ at the final follow-up (> 12 months).

### Radiographic measurements

Two independent observers (both senior spinal surgeons), who did not participate in the operations, performed the imaging analyses, Digital X-ray images were analysed 6 months and 1 year following the operations and during the final out-patient visit to evaluate bone fusion status. The fusion status was classified according to the modified Bridwell criteria^22^.

To evaluate the effect of stress on internal fixation and the maintenance of sagittal balance, we measured the Cobb angle using the cephalic and caudal endplates of the infected vertebral body. The changes in the sagittal Cobb angle were calculated pre-operatively, immediately after the operation, and at the final follow-up.

### Statistical analysis

Clinical outcomes and radiographic data are presented as means ± standard deviations and were compared between the PS and TS groups using the Student t-test. The chi-square test was used to compare the incidence of complications and satisfaction levels between the PS and TS groups. The rank-sum test was used to compare the early fusion rates. The Wilcoxon signed-rank test was used to compare the preoperative-postoperative neurological functional outcomes of the two groups separately. P < 0.05 was considered to indicate statistical significance. All analyses were performed using the SPSS 16.0 statistical software package (Statistical Software for Social Sciences, Chicago, IL, USA).

### Ethics approval and consent to participate

This study was approved by the ethical review committee of The First Affiliated Hospital of Nanchang University. All procedures were performed in accordance with relevant guidelines. University before the initiation of the study and a signed informed consent form was obtained from each subject.

## Results

Of the 59 eligible patients, 12 were excluded from the study. Nine patients were lost during out-patient or telephone follow-up, and three died of other diseases during the follow-up period. In total, 47 patients were included for analysis—21 patients with PS and 26 patients with TS.

### Patient demographic data

There were no significant differences in age, sex, body mass index, smoking history, or duration of the follow-up time between the PS and TS groups. Details of the infections and co-morbidities are shown in Tables 1, 2.

### Clinical outcomes of the surgical treatment

The hospitalisation and ICU durations in the PS group were significantly shorter than those in TS group (P < 0.05). In addition, the amount of intraoperative blood loss and suspended erythrocyte transfusion, and the operation time in the TS group were higher than the PS group, but there was no significant difference between the two groups (P > 0.05). A complete listing of the clinical variables for both groups is provided in Table 3.

**Table 3:** Comparison of clinical and radiological parameters in the PS and TS groups.

Parameters	PS	TS	*P* value
Clinical			
Length of hospitalisation (days)	32.48 ± 8.07	40.88 ± 13.52	0.007*
Blood loss during operation (ml)	526.19 ± 197.24	634.62 ± 269.36	0.065
Infusion of suspended red blood cells (u)	1.29 ± 1.42	1.57 ± 1.84	0.536
Operation time (min)	172.38 ± 42.46	190.85 ± 43.86	0.169
Intensive care unit length of stay (days)	2.24 ± 1.04	3.31 ± 1.43	0.003*
Radiographic			
Pre-operative (°)	24.54 ± 5.05	26.62 ± 4.55	0.072
Post-operative (°)	15.59 ± 5.32	15.73 ± 3.97	0.459
Final follow-up (°)	18.48 ± 5.28	18.78 ± 3.80	0.409
Loss angle of correction (°)	2.89 ± 1.42	3.05 ± 1.99	0.376

*PS* pyogenic spondylodiscitis, *TS* tuberculous spondylodiscitis, *ICU* intensive care unit.

The “*” represents significant difference (P < 0.05).

### Post-operative complications

The total incidence of postoperative complications in both groups was 44.7%. Only the number of patients with complications was counted, and there was no overlap if the same patient experienced multiple complication. The complication rate was 42.9% in the PS group and 46.1% in the TS group. There was no significant difference between the two groups (P > 0.05).

In the PS group, two patients experienced cerebrospinal fluid leakage post-operatively. The drainage tubes were removed, re-sutured and healed without serious consequences after intensive anti-infective treatment. One patient developed incision infection in the iliac bone removal area, which healed after changing the dressing and the administration of long-term antibiotics. One patient was followed up for 6 months with a pedicle screw fracture without any discomfort. One patient showed weakness during right ankle extension. However, the symptoms gradually improved within six months post-operatively. Pulmonary infection occurred in two cases and deep venous thrombosis occurred in one case post-operatively.

In TS group, cerebrospinal fluid leakage was found in two cases. The tube was extubated 48 h later; however, there was still cerebrospinal fluid outflow from the orifice of the drainage tube, which was improved after silk blood suturing. One case developed a deep wound infection, which healed following debridement, suturing and active anti-infective treatment. One patient was found to have pedicle screw dislocation penetrating the spinal canal, but there were no neurological symptoms. Pulmonary infection occurred in three cases. No other serious complications were caused by active anti-inflammatory treatment. One case developed heart failure, which was cured after proper treatment. Drug induced liver damage occurred in two patients, which improved following treatment. One patient experienced a local recurrence of tuberculosis infection, which was controlled after secondary debridement. Deep venous thrombosis of the lower extremities was found in one patient post-operatively and improved after treatment. Lacunar cerebral infarction occurred in one patient, but there was no dysfunction.

### Related outcomes of the clinical efficacy of surgical treatment

The neurological status of the patients is shown in Table 4. All the patients in both groups had different degrees of neurological symptoms prior to their operations. According to the ASIA classification, there were eight cases of ASIA grade D, 12 cases of grade C, and one case of grade B in the PS group, and nine cases of ASIA grade D, 15 cases of grade C, and two cases of grade B in the TS group. At the final follow-up, there were three cases of ASIA grade D and 18 cases of grade E in the PS group. In the TS group, there were four cases of ASIA grade D and 22 cases of grade E. There were no intra-operative nerve injuries in either group. Our results showed that the neurological symptoms of all the patients had improved post-operatively (P < 0.001).

**Table 4:** Neurological recovery according to the American Spinal Injury Association (ASIA) impairment scale.

Pre-operation	Group PS/TS	Final follow-up in the PS group					Final follow-up in the TS group				
		A	B	C	D	E	A	B	C	D	E
A	0/0										
B	1/2				1					1	1
C	12/15				2	10				3	12
D	8/9					8					9
E											

*PS* pyogenic spondylodiscitis, *TS* tuberculous spondylodiscitis.

The final follow-up time is more than 12 months.

The Wilcoxon signed-rank test was used to compare the pre-operation and final follow-up neurological functional outcomes of the two groups separately (P < 0.001 in PS, P < 0.001 in TS).

**Figure 1:**
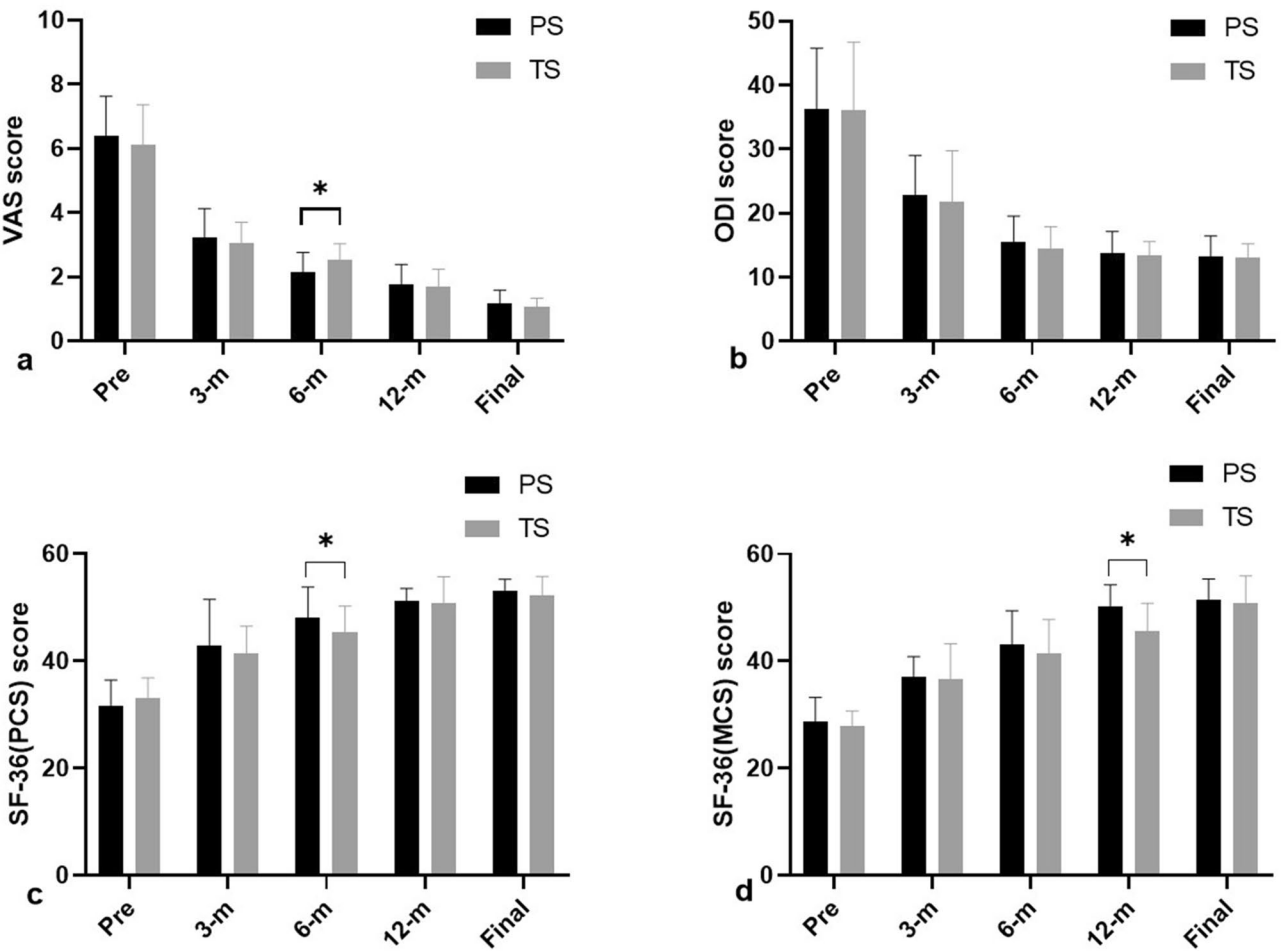
Outcomes of the clinical efficacy in the two groups as measured by visual analog scale (VAS), Oswestry disability index (ODI), and short form 36 survey ((SF-36) scores at pre-operative, 1-month (1-m), 3-month (3-m), 6-month (6-m), and final follow-up (Final) after operation. The histogram show (**a**) VAS, (**b**) ODI, (**c**) SF-36 (physical component summary, PCS), (**d**) SF-36 (mental component summary, MCS) scores between the pyogenic spondylodiscitis (PS) and tuberculous spondylodiscitis (TS) at different follow-up time points.The “*” represents significant difference (P < 0.05).

During follow-up period, the VAS, ODI, and the mental component summary (MCS) score and physical component summary (PCS) score of the SF-36 in the PS and TS groups were significantly improved post-operatively (Fig. 1, Table 5). At the six-month follow-up, the VAS score of the PS group (2.15 ± 0.61) was significantly (P < 0.05) better than the TS group (2.52 ± 0.51) (Fig. 1a). For the ODI and PCS scores, the TS group had slightly worse scores than the PS group (Fig. 1b,c). However, the average PCS score in the PS group was 48.19 ± 5.63, which was significantly better than the TS group (45.42 ± 4.87) at the 6-month follow-up (P < 0.05) (Fig. 1c). One year post-operatively, the average MCS score in the PS group was 50.19 ± 4.07, which was better than the TS group (45.62 ± 5.22), and the differences were statistically significant (P < 0.05) (Fig. 1d). Approximately 83% of patients reported satisfactory results based on the modified MacNab standard.

**Table 5:** Comparison of the ODI, VAS and SF-36 scores between PS and TS.

Parameter	Preoperative	3-months	6 months	1-year	Final
VAS-back, mean ± SD					
PS	6.38 ± 1.24	3.24 ± 0.89	2.15 ± 0.61	1.76 ± 0.63	1.19 ± 0.40
TS	6.12 ± 1.24	3.04 ± 0.66	2.52 ± 0.51	1.69 ± 0.55	1.07 ± 0.27
P value	0.235	0.191	**0.016**	0.343	0.128
ODI, mean ± SD					
PS	36.29 ± 9.46	22.76 ± 6.26	15.43 ± 4.15	13.76 ± 3.37	13.19 ± 3.24
TS	36.08 ± 10.63	21.77 ± 8.01	14.46 ± 3.43	13.38 ± 2.17	13.04 ± 2.19
P value	0.472	0.322	0.193	0.322	0.423
SF-36 (PCS), mean ± SD					
PS	31.71 ± 4.77	42.81 ± 8.72	48.19 ± 5.63	51.33 ± 2.24	53.09 ± 2.19
TS	33.12 ± 3.74	41.38 ± 5.13	45.42 ± 4.87	50.88 ± 4.89	52.35 ± 3.47
P value	0.132	0.244	**0.038**	0.349	0.197
SF-36 (MCS), mean ± SD					
PS	28.67 ± 4.53	37.05 ± 3.77	43.19 ± 6.22	50.19 ± 4.07	51.52 ± 3.83
TS	27.81 ± 2.86	36.69 ± 6.58	41.50 ± 6.28	45.62 ± 5.22	50.84 ± 5.06
P value	0.216	0.414	0.181	**0.000**	0.307

Significant values are in bold.

*PS* Pyogenic spondylodiscitis, *TS* tuberculous spondylodiscitis, *VAS* visual analog scale, *ODI* Oswestry Disability Index, *PCS* phyisical component summary scale, *MCS* mental component summary scale, *SD* standard deviation.

### Imaging outcomes of surgical treatment

The average Cobb angle measurements of the PS group pre-operatively, post-operatively, and at the final follow-up were 24.54 ± 5.05°, 15.59 ± 5.32°, and 18.48 ± 5.28, respectively. For the TS group, the average pre-operative, post-operative, and final follow-up Cobb angle measurements were 26.62 ± 4.55°, 15.73 ± 3.97°, and 18.78 ± 3.80°, respectively. At the final follow-up, the mean loss angle of correction was 2.89 ± 1.42° and 3.05 ± 1.99° in the PS and TS groups, respectively. No statistical difference between the two groups was found. The Cobb angle was corrected operatively in both groups, but there was no statistical significance between the two groups at the pre-operative, post-operative, or final follow-up periods (Table 3).

**Figure 2:**
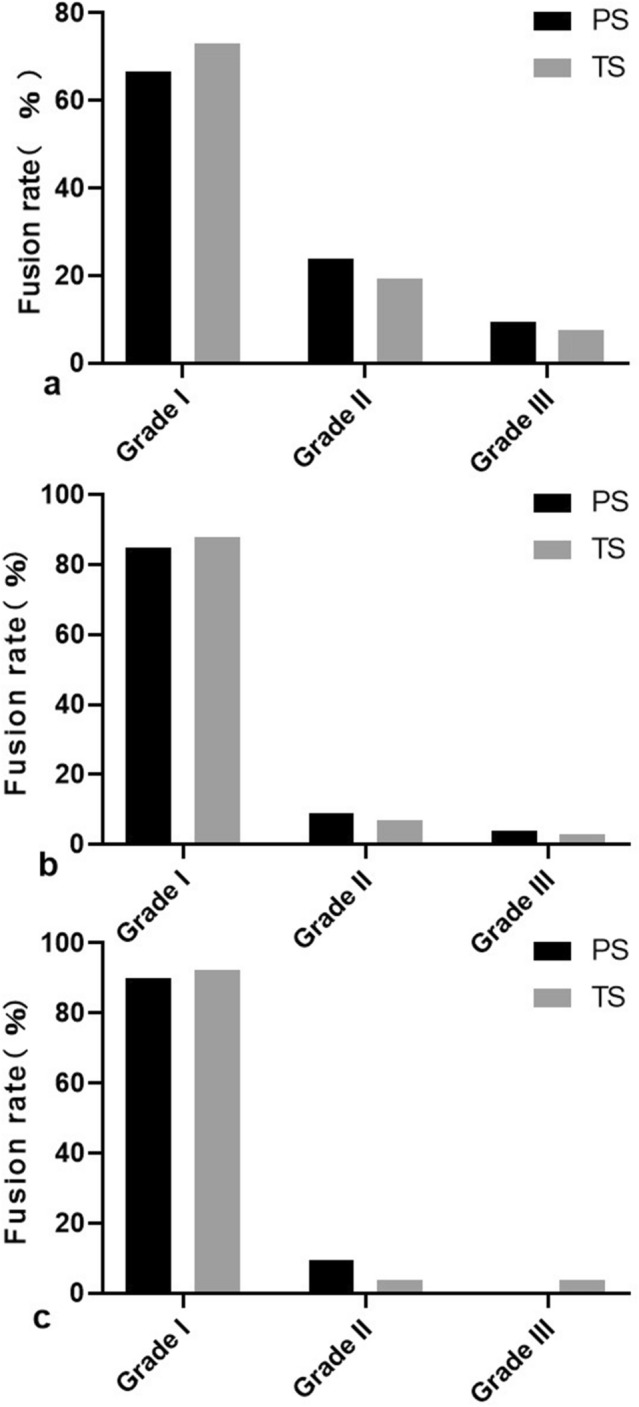
Post-operative fusion rates in the pyogenic spondylodiscitis (PS) and tuberculous spondylodiscitis (TS) groups at different follow-up time points. Follow-up time at 6 months (**a**), at 12 months (**b**), more than 12 months (**c**). Fusion rates are shown as Bridwell et al. described interbody fusion rating, with grade 1 was considered solid fusion, which was compared PS and TS groups with interbody fusion using rank sum test.

At the 6-month, 1-year, and final follow-up time points, the fusion rates of the PS group were 66.7%, 85.7%, and 90.5%, respectively, which were not significantly different from the fusion rates of the TS group (73.1%, 88.5%, and 92.4%, respectively) (Fig. 2).

### Clinical presentations

#### Case 16 of PS group

**Figure 3:**
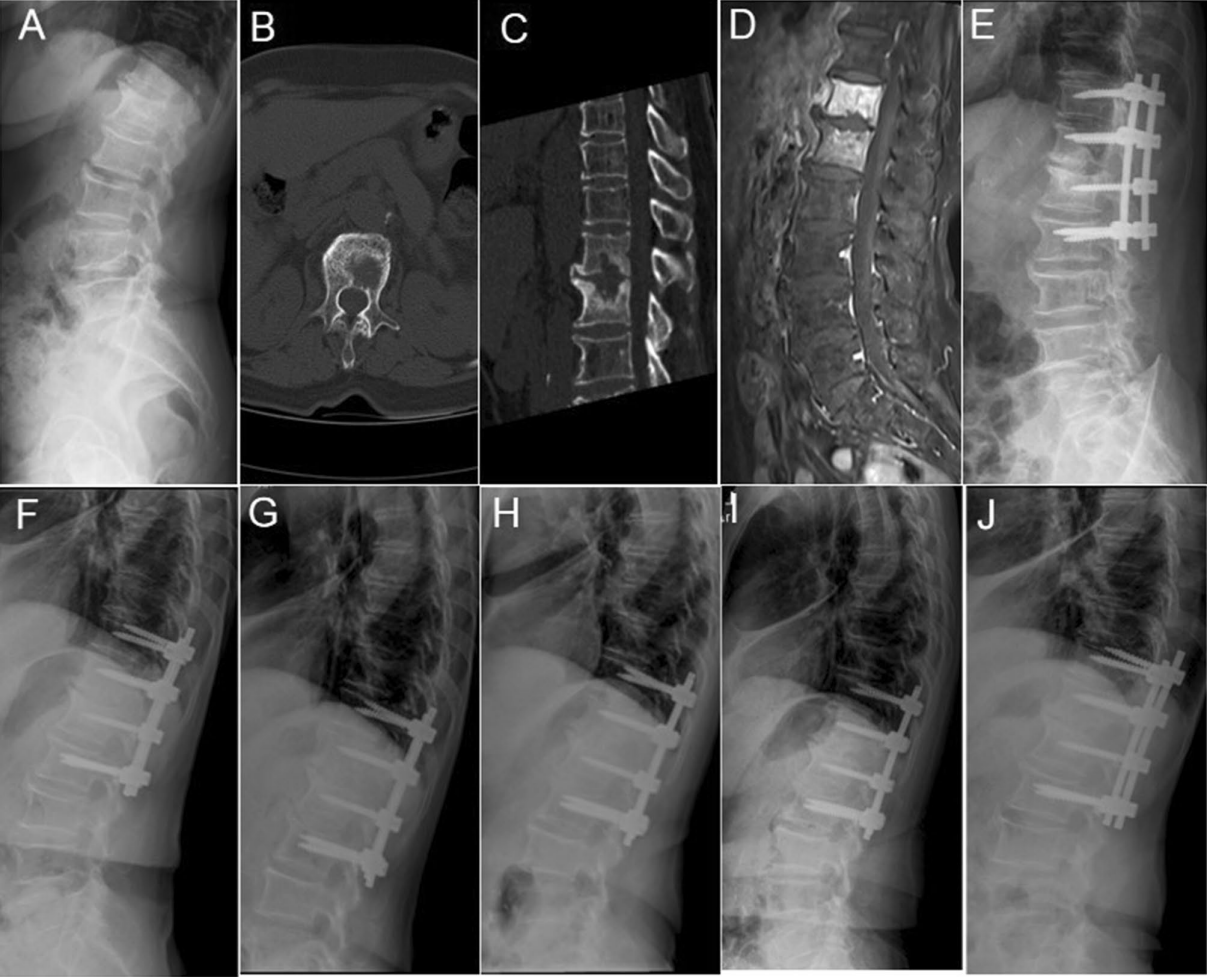
An X-ray revealed T12-L1 disc space narrowing with end-plate destruction (**a**); Computed tomography and magnetic resonance imaging (**b**–**d**) show vertebral bone destruction and paravertebral abscess formation. X-rays taken at 3 days, 3 months, 6 months, 1 year, and 2 years post-operatively, and at the final follow up, respectively (**e**–**j**).

A 72-year-old male who experienced pyogenic infection complained of severe lower back pain, with diabetes, renal failure and hepatocellular carcinoma. An X-ray showed narrowing of the intervertebral disc space in T12-L1 (Fig. 3a) and a CT scan showed bone destruction in T12-L1 with obvious hyper osteogeny and sclerosis (Fig. 3b,c). A lateral magnetic resonance imaging (MRI) on T2 showed high signal intensity of the T12-L1 vertebral body, loss of the intervertebral disc and irregular destruction of the vertebral endplate boundary (Fig. 3d). She received conservative treatment for one month and, although the pain lessened, the pain and inflammation remained. As a result, she underwent one-stage posterior operation. Following the operation, her severe lower back pain was significantly improved (Fig. 3e). The specimen extracted during the operation was positive for *S. Aureus*. Lateral X-ray films taken 3 months, 6 months, 1 year, 2 years and at the final follow-up showed that the physiological curvature and intervertebral height of the lumbar vertebrae had gradually recovered, the bone graft had gradually fused and she no longer experienced back pain (Fig. 3f–j).

#### Case 22 of TS group

**Figure 4:**
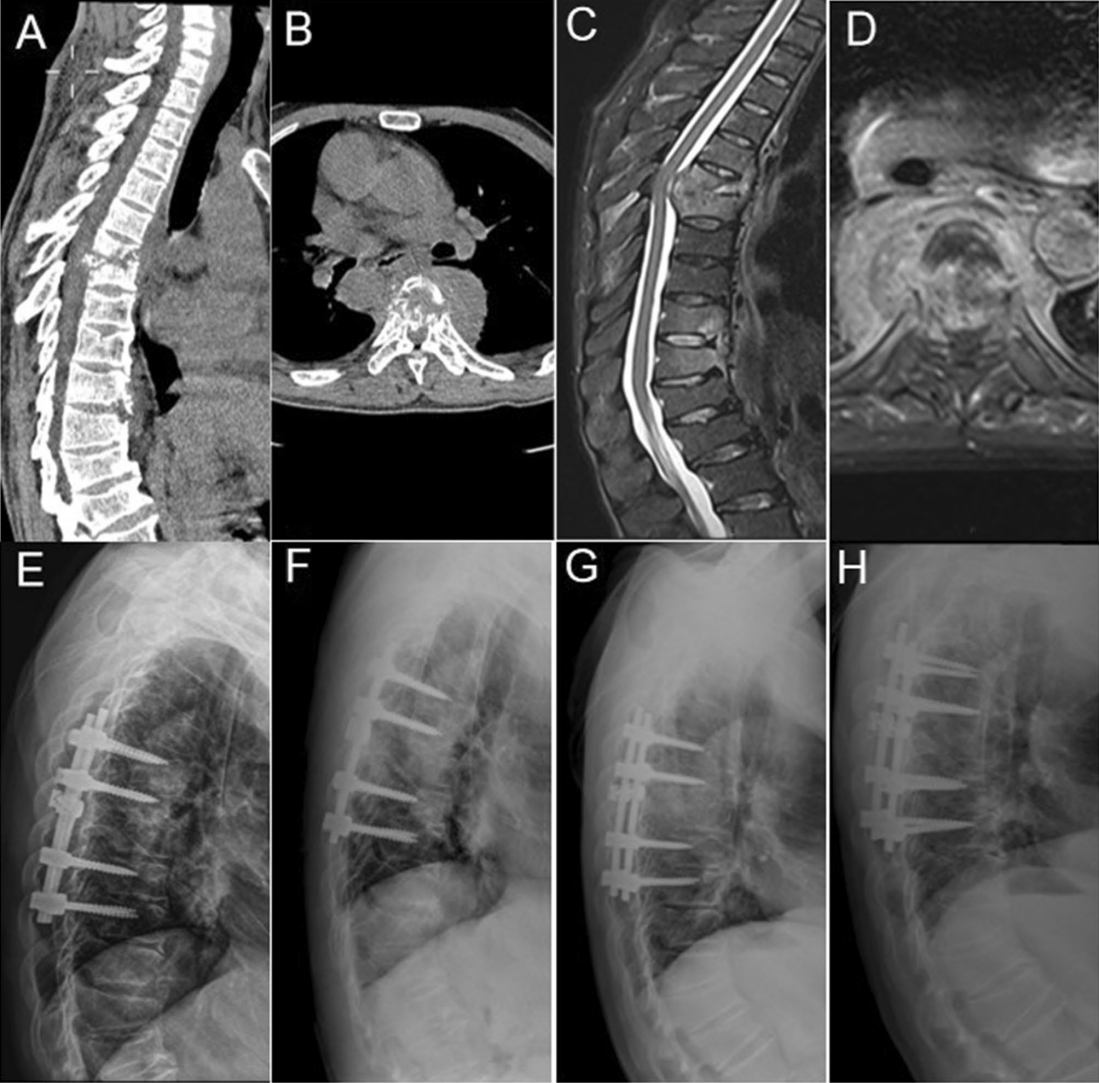
Computed tomography and magnetic resonance imaging (**a**–**d**) show T6–T7 vertebral bone destruction, paravertebral abscess formation and spinal cord compression. X-rays taken 3 days post-operatively (**e**); 6 months post-operatively (**f**); 12 months post-operatively (**g**), and; 24 months post-operatively (**h**).

A 76-year-old male who experienced tuberculosis infection complained of severe lower back pain with neurological symptoms. The patient also had high blood pressure, diabetes, and bladder cancer. A CT scan and MRI showed bone destruction, para-vertebral abscess formation and spinal cord compression in T7–T8 (Fig. 4a–d). Following three months of medication and brace treatment, he underwent operation to reduce the severity of the back pain (Fig. 4e). The specimen extracted during the operation was positive for *M. tuberculosis*. Lateral X-ray films at 6 months, 1 year and 2 years post-operation showed that the bone graft had gradually fused (Fig. 4f–h).

## Discussion

The purpose of the surgical treatment for thoracolumbar infectious spondylitis in the elderly is to facilitate early mobilization after the operation. One-stage posterior focus debridement, bone graft fusion, and internal fixation are widely accepted methods for the treatment of spinal infections in the elderly^10,23^. For elderly patients in poor health and with poor surgical tolerance, this method can effectively improve the safety of the operation. Our results show that the surgical treatment of infectious spondylitis was effective, enabling 91% of the patients who received surgical treatment to walk independently during the follow-up period. Although we only included patients over 65 years of age, our results are consistent with previous reports that surgical treatment of infectious spondylitis has good clinical and radiological outcomes^24–27^. For example, Okada et al.^27^ compared the clinical and radiological results of surgery for pyogenic and tuberculous spondylitis, and they found that the outcome of surgical treatment was favorable for both PS and TS, with no difference in neurological recovery between the PS and TS groups; However, the length of hospital stay of TS patients was significantly longer than that of PS patients, and there was no difference in correction rate and loss of correction rate among different disease types.

In our study, we found no significant differences between the PS and TS groups for mean blood loss and the operation duration. However, hospitalization and ICU stay duration in the PS group were significantly shorter than in the TS group. Okada et al.^27^ reported similar results. TS is a long-term chronic wasting disease that is usually associated with systemic diseases such as tuberculosis, which may help to explain this phenomenon^28^. Thus, a post-operative stay in the ICU and delays in the recovery process would be inevitable. In particular, the onset of tuberculosis is typically unknown, the early symptoms are atypical and it takes longer to diagnose, which partially explains why the hospitalisation duration for patients in the TS group was longer than in the PS group. In our study, 44.7% of patients had post-operative complications. In a related study of elderly patients with spinal tuberculosis, Luo et al.^29^ reported a 97% incidence of complications, which may be due to the absence of statistics on the complications of water-electrolyte imbalance. However, we did not find significant differences in complication rates between the two groups.

In terms of the clinical follow-up results, the VAS, ODI and the MCS and PCS scores of the SF-36 for the TS and PS groups were significantly improved from the pre-operative evaluation to the final follow-up. However, the VAS score for lower back pain in the TS group was significantly lower than that in PS group six months post-operatively (P < 0.05) (Fig. 1, Table 5). Okada et al.^27^ reported that the duration of achieving a CRP-negative result in a TS group was longer than for a PS group (105.9 ± 16.3 vs 52.6 ± 20.2 days), and patients with tuberculous spondylitis experienced more severe inflammatory stimulation within a short period following an operation. Therefore, before inflammation subsides, PCS scores may demonstrate significant differences. Correspondingly, the PCS scores in our TS group were lower than the PS group at the 6-month follow-up. At the 1-year follow-up, the MCS score of the TS group was worse than the PS group.

The literature^30^ suggests that tuberculosis is usually associated with poor living standards and social factors, which can more strongly affect the elderly. Therefore, we speculate that surgical trauma has a greater impact on patients with TS than PS; although, the difference may become indistinct over time. Chun Kim et al.^31^ reported that at the final follow-up of 485 people, 85% demonstrated excellent or good results in lumbar fusion in the elderly according to the MacNab’s criteria. Similarly, approximately 83% of the patients in our study were satisfied with the post-operative results, indicating that the positive effects of surgical treatment for the elderly should be expected if the operation is safe.

Similar results from other studies^32–35^ have shown that solid bone fusion occurs in over 90% of cases. Likewise, the solid fusion rates in the PS and TS groups in our study also reached 90.5% and 92.4%, respectively, at the final follow-up. Although there was no statistical difference between the PS and TS groups, both achieved good fusion rate results. We assumed TS might hamper the maintenance of alignment due to necrotic disease and osteoporosis^27,29^, and it has been reported that TS is more frequently associated with greater deformity than PS^36^. However, we found no significant difference in the correcting angle loss between the PS and TS groups. This is similar to that reported by Okada et al.^27^, however, definitive results may require longer follow-up observation times.

There were some limitations in our study. First, the sample sizes in the two groups were small. A sample size of 47 patients is insufficient and was due to the small number of elderly surgical patients. Second, our average follow-up period of 26 months was not enough to observe long-term effects. Finally, our study focused on specific sub-groups and did not make comparisons with conservative treatments in the elderly, which may have led to inaccurate results. Further, multi-centre, randomised, long-term follow-up studies are needed to overcome these issues.

## Conclusion

Our one-stage posterior debridement, decompression, interbody fusion, and internal fixation can be considered a safe and effective method of treating spinal infections in the elderly. This method can improve nerve function, reconstruct spinal stability, and enhance the quality of life of elderly patients. The hospitalization time and ICU time of TS group were significantly prolonged, but the clinical effect was similar to that of PS group in the long term (more than 1 year).

## Data Availability

The datasets supporting the conclusions of this article are included within the article. The raw data can be requested from the corresponding author on reasonable request.

## Abbreviations

PS: Pyogenic spondylodiscitis
TS: Tuberculous spondylitis
ASIA: American Spinal Injury Association
VAS: Visual analog scale
ODI: Oswestry disability index
SF-36: Short form-36 health survey questionnaire
PCS: Physical component summary
MCS: Mental component summary
ICU: Intensive Care Unit
